# Extracellular matrix dynamics: tracking in biological systems and their implications

**DOI:** 10.1186/s13036-022-00292-x

**Published:** 2022-05-30

**Authors:** Michael Hu, Zihan Ling, Xi Ren

**Affiliations:** Department of Biomedical Engineering, Carnegie Mellon University, 5000 Forbes Avenue, Pittsburgh, PA 15213 USA

**Keywords:** Extracellular matrix (ECM), Lung, Proteomics, Newly synthesized protein, Stable isotope labeling by amino acids in cell culture (SILAC), Bioorthogonal non-canonical amino acid tagging (BONCAT), Bioprinting

## Abstract

The extracellular matrix (ECM) constitutes the main acellular microenvironment of cells in almost all tissues and organs. The ECM not only provides mechanical support, but also mediates numerous biochemical interactions to guide cell survival, proliferation, differentiation, and migration. Thus, better understanding the everchanging temporal and spatial shifts in ECM composition and structure – the ECM dynamics – will provide fundamental insight regarding extracellular regulation of tissue homeostasis and how tissue states transition from one to another during diverse pathophysiological processes. This review outlines the mechanisms mediating ECM-cell interactions and highlights how changes in the ECM modulate tissue development and disease progression, using the lung as the primary model organ. We then discuss existing methodologies for revealing ECM compositional dynamics, with a particular focus on tracking newly synthesized ECM proteins. Finally, we discuss the ramifications ECM dynamics have on tissue engineering and how to implement spatial and temporal specific extracellular microenvironments into bioengineered tissues. Overall, this review communicates the current capabilities for studying native ECM dynamics and delineates new research directions in discovering and implementing ECM dynamics to push the frontier forward.

## Introduction

The extracellular matrix (ECM) describes the noncellular network of macromolecules that is present in almost all biological tissues. Primarily composed of fibrous proteins and proteoglycans, the ECM functions to provide mechanical and biochemical support to cells within tissues, and helps drive key cellular events such as differentiation, migration, and proliferation [1]. The composition of ECM overall and how it varies in specific tissues and organs has been extensively reviewed [1–4]. However, few have focused on the changes in ECM composition over time: the transitional ECM differences between tissue states or between healthy homeostasis to pathological progression. These changes stem from the synthesis, modification, and degradation of ECM biomolecules, in particular the protein components of the ECM, and are collectively referred to as ECM dynamics. ECM dynamics regulate cellular activities and affects tissue properties and functions; understanding these dynamic changes of the ECM is crucial to bettering our understanding of tissue pathophysiology and boosting our ability to engineer functional biological systems. In this review, we use the lung as the primary model organ to highlight what is changing in the ECM, its significance to the surrounding cells, how to measure these changes, and the implications these changes have for tissue engineering.

### The ECM and its dynamics

The ECM is generally composed of fibrous proteins for the major structural framework and heavily glycosylated proteoglycans for signaling and regulation purposes [1, 5–7]. Various proteins, such as collagens and elastin, are synthesized as monomers in cells, post-translationally modified in the Golgi, packaged and released via secretory vesicles, and assembled into macromolecules in the extracellular space [8–13]. The resulting macromolecules commonly form fiber structures, such as elastic fibers - formed from elastin combining with glycoproteins like fibrillins and fibulins [14–16]- and fibrillar collagens like collagens I, II, III, V, and XI [17]. However, non-fibrous supramolecular structures are also assembled, as seen with the nonfibrillar collagen IV (lateral networks) and laminin (branched structures) [17–21]. These macromolecules are then crosslinked to one another by enzymes such as lysyl oxidase (LOX) to achieve desired mechanical properties and establish organ-specific tissue architectures [22–24]. For example, collagen IV and laminin are crosslinked to form the main architecture of the thin ECM surrounding cells, also known as basement membranes, while the fibrous proteins form 3D networks in the interstitial spaces [19, 20, 25–28]. These structural proteins coordinate with each other to deliver distinct mechanical characteristics under different stress levels. Taking the lung parenchyma as an example: at low stress levels, elastic fibers are the main load-bearing component, while at high stress levels the stiffer collagen fibers take over the load-bearing function, stiffening the tissue [4, 29, 30]. This 3D network also acts as a scaffold providing anchor points for other ECM proteins and cells to adhere to, allowing for transduction of extracellular mechanical forces such as stress and strain into signals for the cells to understand. Pulmonary models have demonstrated matrix stiffening mediating actin signaling to promote myofibroblast differentiation [31–33], and mesenchymal stem cells are known to change their morphology in response to the stiffness of the ECM that they are anchored to, transitioning their phenotypes from neuron-like, to myoblast-like, and to osteoblast-like with increasing matrix stiffness [34].

Proteoglycans, the other major component of the ECM, are each composed of a core protein with glycosaminoglycans (GAGs) attached and can be classified into multiple subgroups. Each subgroup matches the GAG type attached to the said proteoglycan: heparan sulfate (HS), chondroitin sulfate (CS), dermatan sulfate (DS), and keratan sulfate (KS) [35]. Proteoglycans can be held together through their interaction with another special type of GAG, hyaluronic acid (HA) [7, 36, 37]. As the largest biomolecule in the ECM, HA serves as a framework that non-covalently binds to the core proteins of proteoglycans, anchors these proteoglycans to the fibrous network, and together regulates ECM signaling [35, 38]. Furthermore, many proteoglycans have the inherent ability to bind growth factors in order to regulate their stability and diffusion within the ECM and facilitate their interactions and signaling with cell surface receptors. For example, HS proteoglycans can facilitate fibroblast growth factor 2 (FGF2), hepatocyte growth factor (HGF), and transforming growth factor beta (TGF-β) signaling by immobilizing them via their heparin-binding domains [39–42]. Proteoglycan binding can also be inhibitory for the growth factor function. For example, decorin, a CS/DS proteoglycan, is reported to bind and inactivate TGF-β to prevent fibril assembly and regulate cell differentiation during the pseudoglandular stage of lung development [35, 38, 43–45]. Proteoglycans play a significant role in tissue health; the removal of these proteoglycans from a decellularized lung ECM scaffold impairs the scaffold’s ability to bind crucial ECM-associated growth factors, resulting in compromised ability to support cellular metabolism and growth [42].

While structural proteins and proteoglycans form the main structural foundation of the ECM, they only account for ~ 26% of ECM-associated genes; the rest of the matrisome – the global ECM and ECM-associated protein set – are associated with various regulatory functions [46, 47]. Regulatory proteins such as ECM-modifying enzymes (e.g., LOXs), cytokines, and growth factors, interact with and remodel the ECM through direct and indirect mechanisms. Together with the structural proteins and proteoglycans, these ECM-associated proteins interface with cells and the ECM itself to reshape the extracellular microenvironment as needed. ECM-cell signaling takes place through many biological mechanisms, such as cellular receptor signaling. To summarize, cell-surface receptors bind to the ECM and its associated biomolecules (growth factors, cytokines, etc.) to regulate key biological events such as cell adhesion, survival, and tissue morphogenesis [48, 49]. These receptors include membrane-embedded proteins such as integrins (activated by fibronectin, vitronectin, collagen, and laminin), growth factor receptors, discoidin domain receptors (activated by various types of collagens), and CD44 (receptor for HA) [50–54]. These ECM-cell interactions are crucial to healthy tissue development. For example, newborn lungs from mutant mice without the a3 integrin subunit displayed decreased branching from the major bronchi and altered epithelial morphology at the terminal respiratory branches from flattened to cuboidal [55]. Cell-surface receptors also engage in crosstalk with each other: integrins are well known for regulating growth factor receptors and vice versa [56]. Epidermal growth factor (EGF) receptors can be activated by integrins in absence of EGF ligand, and vascular endothelial growth factor (VEGF) can activate αVβ3, αVβ5, α5β1, and α2β1 integrins via the VEGFR2 receptor, affecting processes such as cell adhesion and migration [57, 58]

ECM-cell receptor signaling can be further regulated by proteolytic cleavage of matrix proteins during ECM remodeling, giving rise to peptide fragments, termed matricryptins, with newly revealed cryptic sites and bioactivities for cellular receptor interactions [49, 59–61]. Proline-glycine-proline (PGP), a tripeptide matricryptin derived from collagen I, is thought to be chemotactic to neutrophils and promote inflammatory responses in lung injury models that ultimately lead to fibrotic transformation [59, 62]. Mechanical force can also reveal cryptic sites; for example, fibronectin contains cryptic sites that can be exposed by tension. These sites are self-associative in nature and allow fibronectin to self-assemble at sites of high tension [63]. Aberrant myofibroblasts are thought to utilize this mechanism and pull on the ECM to stiffen tissues, resulting in fibrogenesis in organs such as the lungs [64].

All these ECM signals facilitate communication between different ECM components and between the ECM and cells to maintain proper tissue architecture and homeostasis. Spatially, the ECM can be divided into two categories: interstitial matrix and pericellular matrix [4, 7, 65]. Interstitial matrix makes up the bulk of ECM and contains most ECM components (collagens, fibronectins, proteoglycans) that are assembled into a GAG-rich matrix [1, 4, 66]. Pericellular matrix describes the ECM immediately surrounding cells, which possesses properties and compositions different from interstitial matrix and unique to the said cells; basement membranes can be considered a form of pericellular matrix unique to endothelial and epithelial cells and is composed of specific ECM components such as laminins, nidogens, perlecan, agrin, and collagen IV [7, 65, 67, 68]. In contrast, the pericellular matrix surrounding tendon cells is composed of collagen VI, versican, and fibrillin-2 [69]. Both interstitial and pericellular ECM are constantly being remodeled, and their properties are determined by the combined action of ECM modulators that keep each other in check. For example, the crosslinking LOXs and LOX-like enzymes are counterbalanced by the proteolytic activities mediated by matrix metalloproteinases (MMPs), which have an additional counterbalance with tissue inhibitors of metalloproteinases (TIMPs) [70–73]. These extracellular enzymes with opposing functions keep each other in balance and generate a matrix environment that is conducive to local cell activities.

However, sometimes it is desirable for there to be changes in matrisomal composition. These compositional ECM dynamics can be temporal or spatial in nature. Temporal differences in ECM composition occurs between different stages of biological processes such as wound healing and tissue development. For example, in lung injury models induced by allergen, bleomycin, surgery, and naphthalene, matrix proteins taking part in the inflammation stage of lung wound healing (MMP2, TIMP1) are distinct from those involved in the subsequent re-epithelization stage (MMP7, MMP14) [74–79]. Similarly, during lung organogenesis, the ECM composition undergoes dynamic changes between each developmental stage to support temporal specific events in tissue morphogenesis and cellular specification [80, 81]. The murine fetal lung ECM consists of more proteoglycans and GAGs compared to that of the adult lung, which correlates with higher activity of ECM signaling for the rapid and massive changes in cellular phenotypes and tissue organization during embryonic lung development [81, 82]. Compared to the adult counterparts, fetal human and murine lungs express higher level of MMP-2 and lower level of TIMP-3, correlating with decreased ECM remodeling as the lung transitions from development to adult homeostasis [81, 83]. Further, elastin production is maximal during the terminal saccular stage of lung development, where elastin helps drive alveolar septation [81, 84].

**Fig. 1:**
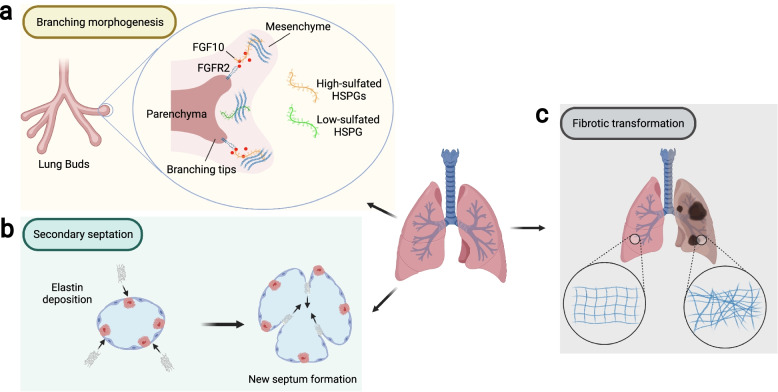
The dynamic ECM microenvironment plays key roles in lung organogenesis and pathogenesis. **a** During murine lung branching morphogenesis, highly sulfated heparin-sulfate proteoglycans (HSPGs) at the mesenchyme surrounding the branching tips act to bind and enrich FGF10 to enable effective activation of FGFR2 on the nearby epithelial cells, promoting epithelial branching towards the desired directions. **b** During the terminal saccular stage of lung development, the selective deposition of elastin around the existing alveoli drives the formation of new alveolar septa, a process termed as secondary septation. **c** During the progression of pulmonary fibrosis, excessive secretion, deposition, and abnormal arrangement of collagen leads to lung malfunction and compromised gas-exchange efficiency

Even within the same biological stage, there exists spatial differences in ECM composition across different organs, in the form of varying component ratios, organizations, and distributions of ECM components, thereby creating extracellular microenvironments with organotypic biological and mechanical properties. For example, elastic tissues like the lung and arteries are generally composed of collagen-and-elastin-rich matrix while the ECM of central neural systems are mostly GAG-rich structures [1, 6, 85–87]. Even within a particular organ, the composition varies spatially across different tissue compartments depending on their architecture and function. The interstitial matrix of the lung parenchyma is rich in type I and III collagen and elastin for contraction purposes while the basement membrane between the alveoli and nearby capillaries is rich in laminin, HA, and proteoglycans to support and facilitate gas exchange [4, 30, 87–89]. During development, spatial patterning of matrisomal proteins in the extracellular space helps guide cell migration and differentiation, leading to the establishment of heterogeneous organ structures. For example, the localization of FGF10 ligands to be adjacent to the branching tips of the nascent airway is critical for proper branching morphogenesis of the respiratory tracts, the process by which bronchi and bronchioles are formed [90]. It has been suggested that this FGF10 patterning is maintained by the heparin sulfates (HS) proteoglycans in the ECM, which store and concentrate FGF10 to be near the branching tips (Fig. 1a) [91, 92]. Similarly, during the process of secondary alveolar septation in lung alveologenesis, areas of high elastin content tend to form ring-like structures on the alveolar epithelium that correspond to the tips of future septa (Fig. 1b) [93, 94].

ECM alterations are usually well-controlled processes to achieve desired outcomes in tissue state transition, followed by reestablishment of homeostasis, but this is not always the case. Due to repetitive injury or aging, the ECM dynamics enter a positive feedback loop that can be detrimental to tissue health, leading to diseases such as fibrosis and cancer. Fibrosis has long been characterized as the excessive accumulation of collagens and other fibrous ECM components (Fig. 1c), while cancer disrupts normal ECM composition and creates its own, malignant extracellular environment [95, 96]. Furthermore, fibrotic ECM alone can induce profibrotic transformation of normal lung cells [97]. Existing tumors can release extracellular signaling molecules that look for and identify susceptible sites elsewhere in the body, and recruit tumor-associated cells (e.g., hematopoietic progenitor cells and macrophages) to remodel these sites’ ECM to facilitate metastasis [98–100]. In both cases, the ECM becomes imbalanced and continues to spiral even further.

### Tracking ECM dynamics

Tracking and understanding ECM dynamics provides fundamental insights regarding how diverse ECM components function during tissue state transitions and help to reveal ECM signatures that correlate with tissue morphogenesis and pathogenesis. Mass spectrometry (MS) based proteomics is a popular approach to study the protein components of tissues, including the ECM. Conventional MS detects proteins with probabilities that are proportional to their abundances. While this is effective when investigating highly abundant protein species such as structural fibrous ECM components (e.g., collagens) or capturing dramatic changes in ECM composition accumulated over long time spans, MS proteomics falls short when the proteins of interest are in low abundance, having a transient expression profile, or both [3, 101]. Such challenges are commonly encountered when analyzing ECM dynamics as the newly produced ECM proteins are usually in extremely low abundance compared to the bulk pre-existing ECM, and sufficient compositional changes may take weeks of accumulation before they can be reliably detected [102, 103]. As a result, proteins in low abundance or with fast-changing kinetics have a high probability to be neglected by conventional proteomic analysis.

**Fig. 2:**
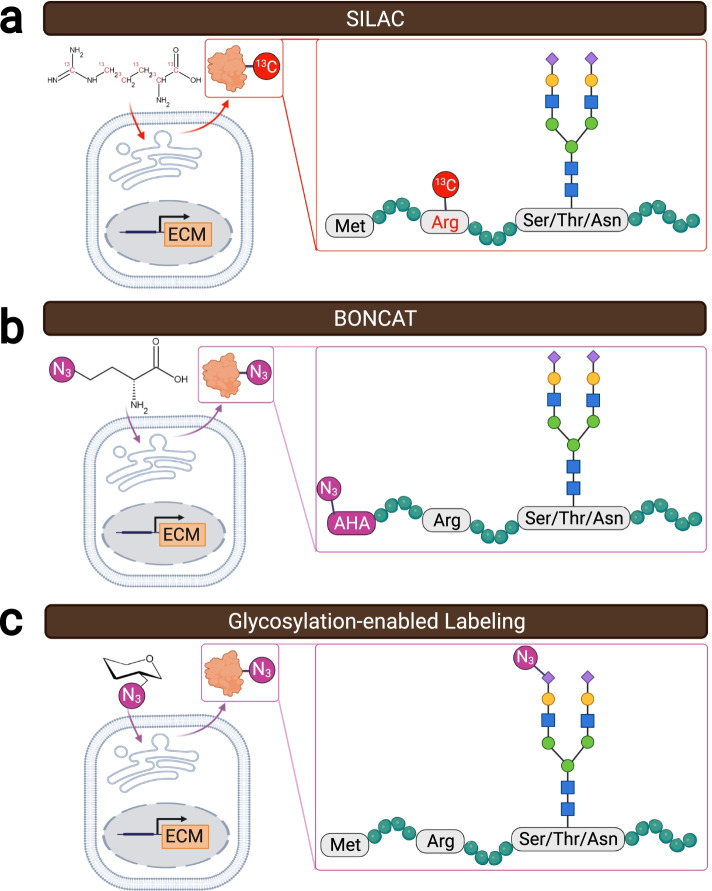
Technologies for labeling NSPs. **a** SILAC incorporates isotopes-labeled amino acids, such as arginine, into NSPs. **b** BONCAT labels NSPs by replacing methionine residues with its azide-bearing analog, such as AHA. **c** Glycosylation-enabled labeling uses azide-bearing monosaccharide probes to tag glycosylated NSPs during post-translational glycosylation

This gap in understanding dynamic ECM changes necessitates technology developments that allow detection and tracking of low-abundance, transient protein species within the organotypic extracellular environment. Metabolic labeling of newly synthesized proteins (NSPs) is an attractive strategy as it allows incorporation of chemoselective tags into new protein additions, including ECM NSPs, during either protein translation or post-translational modification, allowing selective proteomic analysis of NSPs. Commonly used metabolic NSP labeling techniques include isotope labeling, bioorthogonal non-canonical amino acid tagging (BONCAT), and glycosylation-enabled NSP labeling (Fig. 2).

**Fig. 3:**
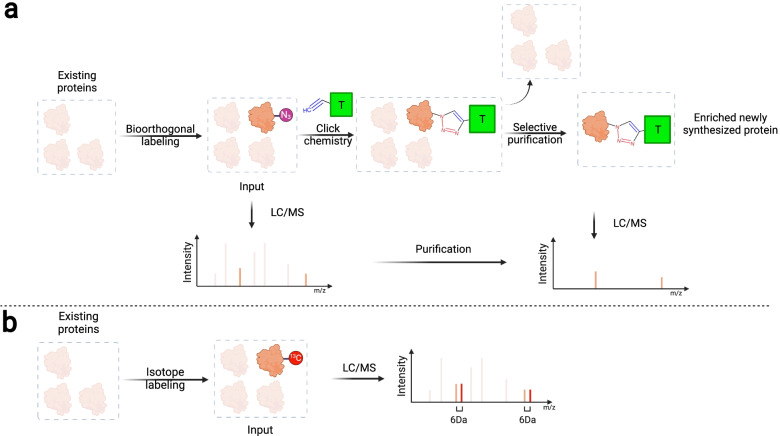
Strategies for proteomic identification of NSPs. **a** Azide-tagged NSPs labeled via bioorthogonal approaches, such as BONCAT and glycosylation-enabled labeling, can be conjugated to affinity tags (e.g. biotin) via the click chemistry, and affinity-purified free from pre-existing proteins to enable ultrasensitive proteomic detection using MS. **b** Isotope-tagged NSPs exhibit a specific molecular weight shift compared to their untagged native counterparts (for example, each.^13^C-Arg tag increases the molecular weight by 6 Da), facilitating the identification of MS peaks corresponding to the labeled NSPs

Isotope labeling of proteins can be accomplished via a variety of methods [101, 104], and a commonly used technique is stable isotope labeling by amino acids in cell culture (SILAC). Briefly, SILAC feeds and incorporates isotope-labeled amino acids, such as arginine, leucine, and lysine, into NSPs produced by cells (Fig. 2a) [105–107]. The resulting isotope-labeled NSP will display a signature mass shift compared to its non-labeled counterpart in MS analysis, and thus facilitate NSP detection (Fig. 3a). However, SILAC has been reported to require a long labeling period (at least 5 days) to label the murine proteome in vivo and does not allow isolation of the isotope-labeled NSPs [108–110]. Nonetheless, recent efforts have validated the feasibility of using SILAC to quantify ECM protein deposition by cells repopulating decellularized human lung scaffold, where temporally specific matrisome changes were observed to correlate with cellular proliferative activity, cell adhesion, and ECM regeneration [111]. Similarly, in a tissue-engineered pulmonary fibrosis model, SILAC facilitated capturing differential expression of matrisomal proteins that underlined increased tissue density, stiffness, and ultimate force, as well as matrix-directed cellular responses in fibroblasts [112].

Bioorthogonal non-canonical amino acid tagging (BONCAT) operates by incorporating non-canonical amino acids (ncAAs) bearing chemoselective tags, such as azide-bearing methionine analog (L-azidohomoalanine, AHA) and alkyne-bearing methionine analog (L-homopropargylglycine, HPG), into the newly synthesized peptide chain during NSP translation (Fig. 2b) [113]. Some ncAAs, such as AHA and HPG, can be processed by endogenous aminoacyl tRNA synthetases (aaRSs) and utilized directly by cells for NSP production. Other ncAAs, such as azidonorleucine (ANL), require mutant aaRSs to be attached to desired tRNAs and subsequently incorporated into NSPs. By controlling the tissue-specific expression of the needed mutant aaRSs, it is possible to track the spatial origin of NSPs using BONCAT, as only the proteins produced by the mutant-aaRS-expressing cells will be labeled [114–116]. The resulting azide/alkyne-labeled NSPs enable effective and selective bioconjugation to desired affinity probes bearing complementary tags via click chemistry, allowing for subsequent affinity purification for the labeled NSPs (Fig. 3b) [113, 117–119]. For this reason, BONCAT has exhibited impressive sensitivity and temporal resolution for NSP detection and has been reported to capture NSPs accumulated over as short as two hours [118]. In the last decade, BONCAT has mainly been used to study the dynamic synthesis of cellular proteins [114, 116, 120–122]. Only recently has BONCAT begun to be implemented for investigating the ECM, such as for visualizing ECM assembly and organization during chondrogenesis [123], for identifying NSP deposition by mesenchymal stem cells in hydrogels [124], and for revealing ECM compositional changes at different stages of mouse development [125, 126]. An emerging trend is to combine BONCAT and SILAC to quantify proteins discovered via BONCAT. In this case, proteins are labeled with both ncAA and isotope labels; the ncAA labels allow for NSP enrichment and thus improved detection sensitivity, while the isotope labels allow for comparative quantification [93, 101, 109, 110].

Unlike SILAC and BONCAT, which directly label the amino acids, glycosylation-enabled labeling utilizes post-translational glycosylation to metabolically label the glycans attached to NSPs (Fig. 2c). Azide- or alkyne-bearing monosaccharide analogs of galactosamine and mannosamine have been developed that specifically label glycosylated NSPs bearing O-linked mucin-type glycans or terminal sialic acid respectively [127–132]. Like BONCAT, once the azide or alkyne tags are incorporated into NSPs via their glycans, subsequent bioconjugation can be performed for visualization or purification purposes (Fig. 3b) [133, 134]. Since glycosylation occurs mainly to membrane-associated and secreted proteins and all ECM proteins are originally secreted by resident cells [135], it will be interesting to examine whether glycosylation-enabled NSP labeling has a labeling preference to matrisomal proteins as compared to SILAC or BONCAT. Future studies should focus on identifying, classifying, and quantifying which proteins are tagged via glycosylation-enabled NSP labeling. Our recent study has validated the use of azido galactosamine analog to label the ECM-NSPs in a wide variety of tissues and organs (such as the lung, heart, liver, kidney, skin, and blood vessel) in vivo as well as during ex vivo lung culture [136]. This ex vivo labeling ability opens up the opportunity to label donor human tissues and thus perform more clinically relevant studies. Further focus on selective analysis of matrisomal NSPs can be achieved when combining proteomic analysis with selective extraction of ECM proteins via decellularization to remove most cellular components. Moreover, glycosylation-enabled labeling can be and has been used to study glycan composition in ECM, i.e., ECM glycomics [37, 137–142].

These above-described metabolic labeling methods can be combined with MS to identify changes in ECM protein synthesis. While this review primarily focuses on ECM compositional dynamics, it is important to note that these methods are not limited to only studying compositional changes. BONCAT and glycosylation-enabled labeling allows fluorophore conjugation to ECM-NSPs to visualize and measure the new additions of ECM architecture in tissues [123, 124]. Furthermore, protein–protein interactions and protein topography (i.e., mapping a protein’s internal structure), topics that are crucial to understanding ECM assembly and architecture, can be studied by modifying BONCAT and using mutant tRNA to insert ncAAs onto pre-determined sites on a protein. The inserted ncAAs can then help identify or alter parts of the protein structure and its interaction with other proteins [114, 115, 143, 144]. We direct the readers to the following reviews for more information on site-specific ncAA labeling and its applications [114, 115, 143].

### Implications of ECM dynamics for ECM engineering

Investigation of ECM dynamics hold implications for biomaterial and tissue engineering by increasing our understanding of native, organotypic ECM compositions, processes, and interactions. Protein patterns discovered through ECM dynamics studies can be recreated in tissue scaffolds to better support or guide cells. These changes may be spatial, temporal, or even spatiotemporal in nature and would require different technologies to capture the nuances of each change. Here, we briefly go over the current states and limits of emerging technologies that would be useful for implementing these ECM changes in engineered tissue morphogenesis.

Spatial ECM patterns, differences, and architecture can be recreated via 3D bioprinting techniques. 3D bioprinting is biofabrication method that allows for the 3D arrangement of cells and biomaterials in a layer-by-layer fashion and has been noted for replicating complex tissue geometries with high precision, such as microfibril muscle structures, entangled vascular networks with oxygen exchange abilities, and neonatal scale whole-heart scaffolds [145–154]. However, these bioprints are generally homogenous in material and printed with compositionally simple bioinks, such as collagen and poly(ethylene glycol) diacrylate (PEGDA), which remain distant from fully recapitulating the complexity of the ECM composition found in native organs. This has not been unnoticed by the field and recent advances in technology development are beginning to allow 3D bioprinting of multiple bioinks, enabling the embedment of multi-material patterns in prints and bringing the resulting constructs closer to replicating spatial ECM heterogeneity [155–158]. Decellularized ECM, from a variety of sources such as the heart, blood vessel, and airway mucosal tissues, has been used recently to generate bioinks with improved tissue-specificity [159, 160]. However, the decellularization process often relies on harsh detergents and has been shown to strip important GAGs and their associated growth factors from the ECM of organs such as the lungs [42]; this gap will need to be addressed to enable better recapitulation of native ECM composition using decellularized ECM bioinks. Investigation regarding ECM dynamics during native tissue morphogenesis holds the potential to discover new important components within the extracellular space, providing critical instruction for formulating more physiologically relevant bioinks for tissue engineering [4, 159].

Apart from bioink formulation, bioprinting also face spatial patterning limitations. Much of native ECM 3D structure remains not completely understood, and without better understanding ECM structure, we are, in a sense, missing blueprints to print from. Common ECM components such as collagens and laminins have had their structures mapped out via techniques such as second harmonic imaging and immunostaining [161–164], but many other ECM protein patterns remain elusive to us. Recent investigations are beginning to bridge these knowledge gaps: decellularization has been used to isolate ECM components with preserved, organ-specific composition and architecture [159, 162–164], various sensor array strategies have been developed for MS and RNA sequencing to discretely divide tissue samples into analyzable “voxels” and spatially track ECM proteomics and transcriptomics [165–167], and nascent matrices have been labeled to study their assembly and growth in hydrogels [123, 168]. However, ECM composition and structure are complex, and it is still unclear which proteins and what structures in the decellularized ECM bioinks result in the reported improved cellular support [159]. Further attention should be directed towards better understanding these proteins and their matrix architectures. Additionally, out of the ECM patterns that we do know, some are too small to be printed; pulmonary capillaries are ~ 7 μm in diameter and basement membranes are 50–100 nm thick, whereas most bioprinting techniques have resolutions in the tens of micrometers [169–171].

ECM composition is not only spatially specific, but also temporally specific. Data from ECM dynamics studies could be used to generate an ECM composition that is specific to a particular stage of organ development or pathological progression, allowing the engineered biomaterials to model temporal snapshots of the tissue of interest. Stage-specific ECM signaling molecules can be introduced to tissue scaffolds via adjustments to the scaffold production chemistries or chemical linkage to existing scaffolds. For example, ECM scaffolds have been functionalized with growth factors such as TGF-β1 and FGF2 to better drive cell differentiation and support cell proliferation respectively [172, 173]. Unfortunately, these scaffolds and scaffolds created by most other ECM biofabrication techniques face a common limitation in that they only capture the ECM composition of one particular stage instead of the evolving ECM microenvironment as is commonly seen in native biological processes. To address this bottleneck, there has been interest in scaffold-incorporable, activable functional groups that can be engineered to respond to outside variables and change the scaffold’s properties, effectively allowing temporal control and even some spatial control over said scaffold properties. This has manifested in scaffolds responsive to multiple stimuli, with photostimulation being the most heavily investigated for tissue engineering purposes [174]. Light has been used to control biochemical cue presentation and mechanical properties in scaffolds through various photochemical strategies such as photocaging, radical polymerization, and crosslink degradation. Photocaging involves adding photosensitive groups to mask the bioactivities of the target biomolecule. After these masked biomolecules are incorporated into scaffolds, light can be shined on them to cleave off the photosensitive group and restore biomolecule functionality [175]. This has been utilized to spatiotemporally control growth factor localization and cell migration [176]. Radical polymerization involves chemistry that allows UV light to generate more radicals for polymerization, increasing the density and stiffness of the scaffold in locations exposed to UV light. A common example of this is methacrylate-based polymerization, which is currently popular in tissue engineering and has been utilized to guide cell differentiation within scaffolds [177]. In contrast, scaffolds can also be made to be photodegradable by making crucial scaffold elements such as crosslinkers photodegradable; this has been utilized to spatiotemporally degrade locations in scaffolds to give room for organoids to develop into and form structures [178, 179]. Apart from light, scaffolds responsive to other stimuli such as ultrasound, magnetic fields, and electric signals have been developed and are actively researched. We recommend the following review for a more in depth look at photo-responsive, ultrasound-responsive, magnetically responsive, and electrically responsive tissue scaffolds [174].

### Concluding remarks

The ECM is a crucial component of all tissues not only as a structural support, but also as a signaling system that communicates environmental cues to cells and influence a wide variety of cellular activities. As tissues change due to development, growth, or disease, the ECM is also being modified to both drive and accommodate said changes. Effective ECM engineering is thus critical for recapitulating the dynamic, evolving extracellular microenvironment necessary for engineered tissue morphogenesis. However, such efforts are hindered by our limited understanding of the ECM dynamics accompanying native biological processes. Much protein profiling has been done to the matrisome, but many current discovery strategies are unable to detect low-abundance, transient protein species. To combat this, new strategies are focusing on the ECM fraction that undergoes active changes such as new ECM synthesis. SILAC has been used to study ECM-NSPs, but its application is limited by its inability to isolate the NSPs from the pre-existing protein background. This challenge is being addressed by the BONCAT technology, which is just beginning to focus on ECM-NSPs, and we look forward to the discoveries that will follow. Glycosylation-enabled labeling has been in literature used to study new ECM accumulation and has recently been demonstrated to work in multiple organs in vivo and ex vivo; efforts to incorporate proteomics with glycosylation-enabled labeling should bear new findings for ECM dynamics. Additionally, just as SILAC and BONCAT have been combined to create a methodology that allows for BONCAT’s purification and SILAC’s quantification steps, glycosylation-enabled labeling could be combine with SILAC to deliver robust quantitative assessment of glycosylated NSPs that is highly prevalent in the matrisome. These research directions will uncover previously unknown ECM proteins that are associated with key developmental or pathological events and map out such event-specific ECM microenvironments spatially and temporally. Apart from protein composition, ECM dynamics studies focused on glycomics, protein–protein interaction, and protein topography would shed further light on additional ECM properties such as 3D structure and biochemical gradients. Extracellular vesicles embedded in ECM have recently been tied to ECM-cell interactions [180–184], and Tenascin-R, a key brain ECM protein, has been observed being endocytosed and resurfacing later, essentially being “recycled” during matrix remodeling [185]. These discoveries bring in additional layers of complexity in understanding how the evolving extracellular microenvironment mediates pathophysiological processes. Knowledge gained from studying these phenomena can then be acted upon via engineered strategies such as 3D bioprinting and ECM functionalization, creating biomimetic ECM conditions to guide cell behaviors and functions.

## Data Availability

N/A.

## Abbreviations

ECM: Extracellular matrix
LOX: Lysyl oxidase
HS: Heparin sulfate
CS: Chondroitin sulfate
DS: Dermatan sulfate
KS: Keratan sulfate
HA: Hyaluronic acid
FGF2: Fibroblast growth factor 2
HGF: Hepatocyte growth factor
TGF-β: Transforming growth factor β
EGF: Epidermal growth factor
VEGF: Vascular epithelial growth factor
PGP: Proline-glycyl-proline
MMP: Matrix metalloproteinase
TIMP: Tissue inhibitors of metalloproteinase
HSPG: Heparin sulfate proteoglycans
MS: Mass spectrometry
NSP: Newly synthesized protein
BONCAT: Bioorthogonal non-canonical amino acid tagging
SILAC: Stable isotope labelling by amino acids in cell culture
ncAA: Non-canonical amino acid
AHA: L-azidohomoalanine
HPG: L-homopropargylglycine
tRNA: Transfer RNA
aaRS: Aminoacyl tRNA synthetase
ANL: Azidonorleucine
PEGDA: Poly(ethylene glycol) diacrylate

## References

[1] Frantz C, Stewart KM, Weaver VM (2010). The extracellular matrix at a glance. J Cell Sci.

[2] Meran L, Baulies A, Li VSW (2017). Intestinal Stem Cell Niche: the extracellular matrix and cellular components. Stem Cells Int.

[3] McKee TJ, Perlman G, Morris M, Komarova SV (2019). Extracellular matrix composition of connective tissues: a systematic review and meta-analysis. Sci Rep.

[4] Tonti OR, Larson H, Lipp SN, Luetkemeyer CM, Makam M, Vargas D (2021). Tissue-specific parameters for the design of ECM-mimetic biomaterials. Acta Biomater.

[5] Rozario T, DeSimone DW (2010). The extracellular matrix in development and morphogenesis: a dynamic view. Dev Biol.

[6] Xing Y, Varghese B, Ling Z, Kar AS, Reinoso Jacome E, Ren X. Extracellular Matrix by Design: Native biomaterial fabrication and functionalization to boost tissue regeneration. Regen Eng Transl Med. 2022;8:55–74.

[7] Theocharis AD, Skandalis SS, Gialeli C, Karamanos NK (2016). Extracellular matrix structure. Adv Drug Deliv Rev.

[8] Muiznieks LD, Keeley FW (2013). Molecular assembly and mechanical properties of the extracellular matrix: a fibrous protein perspective. Biochim Biophys Acta.

[9] Mithieux SM, Wise SG, Weiss AS (2013). Tropoelastin — a multifaceted naturally smart material. Adv Drug Deliv Rev.

[10] Yeo GC, Keeley FW, Weiss AS (2011). Coacervation of tropoelastin. Adv Coll Interface Sci.

[11] Hinek A, Rabinovitch M (1994). 67-kD elastin-binding protein is a protective companion of extracellular insoluble elastin and intracellular tropoelastin. J Cell Biol.

[12] Gelse K, Pöschl E, Aigner T (2003). Collagens–structure, function, and biosynthesis. Adv Drug Deliv Rev.

[13] Weihermann AC, Lorencini M, Brohem CA, de Carvalho CM (2017). Elastin structure and its involvement in skin photoageing. Int J Cosmet Sci.

[14] Kielty CM, Sherratt MJ, Marson A, Baldock C (2005). Fibrillin microfibrils. Adv Protein Chem.

[15] Papke CL, Yanagisawa H (2014). Fibulin-4 and fibulin-5 in elastogenesis and beyond: Insights from mouse and human studies. Matrix Biol.

[16] Kielty CM, Sherratt MJ, Shuttleworth CA (2002). Elastic fibres. J Cell Sci.

[17] Hulmes DJS (2008). Collagen Diversity, Synthesis and Assembly Collagen.

[18] Chute M, Aujla P, Jana S, Kassiri Z (2019). The non-fibrillar side of fibrosis: contribution of the basement membrane, proteoglycans, and glycoproteins to myocardial fibrosis. J Cardiovasc Dev Dis.

[19] Yurchenco PD, Ruben GC (1987). Basement membrane structure in situ: evidence for lateral associations in the type IV collagen network. J Cell Biol.

[20] Yurchenco PD, Furthmayr H (1984). Self-assembly of basement membrane collagen. Biochemistry.

[21] Aumailley M, Bruckner-Tuderman L, Carter WG, Deutzmann R, Edgar D, Ekblom P (2005). A simplified laminin nomenclature. Matrix Biol.

[22] Halper J, Kjaer M (2014). Basic components of connective tissues and extracellular matrix: elastin, fibrillin, fibulins, fibrinogen, fibronectin, laminin, tenascins and thrombospondins. Adv Exp Med Biol.

[23] Rock MJ, Cain SA, Freeman LJ, Morgan A, Mellody K, Marson A (2004). Molecular basis of elastic fiber formation. critical interactions and a tropoelastin-fibrillin-1 cross-link. J Biol Chem.

[24] Rucker RB, Kosonen T, Clegg MS, Mitchell AE, Rucker BR, Uriu-Hare JY (1998). Copper, lysyl oxidase, and extracellular matrix protein cross-linking. Am J Clin Nutr.

[25] Yurchenco PD, Schittny JC (1990). Molecular architecture of basement membranes. The FASEB Journal.

[26] Bosman FT, Stamenkovic I (2003). Functional structure and composition of the extracellular matrix. J Pathol.

[27] Martin GR, Timpl R (1987). Laminin and other basement membrane components. Annu Rev Cell Biol.

[28] Kruegel J, Miosge N (2010). Basement membrane components are key players in specialized extracellular matrices. Cell Mol Life Sci.

[29] Faffe DS, Zin WA (2009). Lung parenchymal mechanics in health and disease. Physiol Rev.

[30] Toshima M, Ohtani Y, Ohtani O (2004). Three-dimensional architecture of elastin and collagen fiber networks in the human and rat lung. Arch Histol Cytol.

[31] Zhao XH, Laschinger C, Arora P, Szászi K, Kapus A, McCulloch CA (2007). Force activates smooth muscle α-actin promoter activity through the Rho signaling pathway. J Cell Sci.

[32] Arora PD, Narani N, McCulloch CAG (1999). The compliance of collagen gels regulates transforming growth factor-β induction of α-smooth muscle actin in fibroblasts. Am J Pathol.

[33] Sandbo N, Lau A, Kach J, Ngam C, Yau D, Dulin NO (2011). Delayed stress fiber formation mediates pulmonary myofibroblast differentiation in response to TGF-β. Am J Physiol Lung Cell.

[34] Engler AJ, Sen S, Sweeney HL, Discher DE (2006). Matrix elasticity directs stem cell lineage specification. Cell.

[35] Schaefer L, Schaefer RM (2010). Proteoglycans: from structural compounds to signaling molecules. Cell Tissue Res.

[36] DeAngelis PL (2012). Glycosaminoglycan polysaccharide biosynthesis and production: today and tomorrow. Appl Microbiol Biotechnol.

[37] Sasisekharan R, Raman R, Prabhakar V (2006). Glycomics approach to structure-function relationships of glycosaminoglycans. Annu Rev Biomed Eng.

[38] Alberts B, Johnson A, Lewis J, Morgan D, Raff M, Roberts K, et al. Cell junctions and the extracellular matrix. Molecular Biology of the Cell (6th edition). New York: Garland Science; 2015. p. 1035–91.

[39] Sterner E, Meli L, Kwon SJ, Dordick JS, Linhardt RJ (2013). FGF-FGFR signaling mediated through glycosaminoglycans in microtiter plate and cell-based microarray platforms. Biochemistry.

[40] Sterner E, Masuko S, Li G, Li L, Green DE, Otto NJ (2014). Fibroblast growth factor-based signaling through synthetic heparan sulfate blocks copolymers studied using high cell density three-dimensional cell printing *. J Biol Chem.

[41] Schultz V, Suflita M, Liu X, Zhang X, Yu Y, Li L (2017). Heparan sulfate domains required for fibroblast growth factor 1 and 2 signaling through fibroblast growth factor receptor 1c *. J Biol Chem.

[42] Uhl FE, Zhang F, Pouliot RA, Uriarte JJ, Rolandsson Enes S, Han X (2020). Functional role of glycosaminoglycans in decellularized lung extracellular matrix. Acta Biomater.

[43] Yamaguchi Y, Mann DM, Ruoslahti E (1990). Negative regulation of transforming growth factor-beta by the proteoglycan decorin. Nature.

[44] Godoy-Guzmán C, San Martin S, Pereda J (2012). Proteoglycan and collagen expression during human air conducting system development. Eur J Histochem.

[45] Kresse H, Schnherr E (2001). Proteoglycans of the extracellular matrix and growth control. J Cell Physiol.

[46] Naba A, Clauser KR, Hoersch S, Liu H, Carr SA, Hynes RO (2012). The matrisome: in silico definition and in vivo characterization by proteomics of normal and tumor extracellular matrices. Mol Cell Proteomics.

[47] Naba A, Clauser KR, Ding H, Whittaker CA, Carr SA, Hynes RO (2016). The extracellular matrix: Tools and insights for the “omics” era. Matrix Biol.

[48] Rosso F, Giordano A, Barbarisi M, Barbarisi A (2004). From Cell–ECM interactions to tissue engineering. J Cell Physiol.

[49] Clause KC, Barker TH (2013). Extracellular matrix signaling in morphogenesis and repair. Curr Opin Biotechnol.

[50] Giancotti FG, Ruoslahti E (1979). Integrin signaling. Science.

[51] Leitinger B (2014). Discoidin domain receptor functions in physiological and pathological conditions. Int Rev Cell Mol Biol.

[52] Ponta H, Sherman L, Herrlich PA (2003). CD44: from adhesion molecules to signalling regulators. Nat Rev Mol Cell Biol.

[53] Harada H, Takahashi M (2007). CD44-dependent intracellular and extracellular catabolism of hyaluronic acid by hyaluronidase-1 and -2. J Biol Chem.

[54] Naor D, Sionov RV, Ish-Shalom D. CD44: Structure, function, and association with the malignant process. Adv Cancer Res. 1997;71:241–319. 10.1016/s0065-230x(08)60101-3 9111868

[55] Kreidberg JA, Donovan MJ, Goldstein SL, Rennke H, Shepherd K, Jones RC (1996). Alpha 3 beta 1 integrin has a crucial role in kidney and lung organogenesis. Development.

[56] Yamada KM, Even-Ram S (2002). Integrin regulation of growth factor receptors. Nat Cell Biol.

[57] Moro L, Venturino M, Bozzo C, Silengo L, Altruda F, Beguinot L (1998). Integrins induce activation of EGF receptor: role in MAP kinase induction and adhesion-dependent cell survival. EMBO J.

[58] Byzova TV, Goldman CK, Pampori N, Thomas KA, Bett A, Shattil SJ (2000). A mechanism for modulation of cellular responses to vegf: activation of the integrins. Mol Cell.

[59] de Castro Brás LE, Frangogiannis NG (2020). Extracellular matrix-derived peptides in tissue remodeling and fibrosis. Matrix Biol.

[60] Whitelock JM, Murdoch AD, Iozzo RV (1996). Underwood PA. the degradation of human endothelial cell-derived perlecan and release of bound basic fibroblast growth factor by stromelysin, collagenase, plasmin, and heparanases. J Biol Chem.

[61] Ricard-Blum S, Salza R (2014). Matricryptins and matrikines: biologically active fragments of the extracellular matrix. Exp Dermatol.

[62] Gaggar A, Jackson PL, Noerager BD, O’Reilly PJ, McQuaid DB, Rowe SM (2008). A novel proteolytic cascade generates an extracellular matrix-derived chemoattractant in chronic neutrophilic inflammation. J Immunol.

[63] Zhong C, Chrzanowska-Wodnicka M, Brown J, Shaub A, Belkin AM, Burridge K (1998). Rho-mediated contractility exposes a cryptic site in fibronectin and induces fibronectin matrix assembly. J Cell Biol.

[64] O’Connor JW, Gomez EW (2014). Biomechanics of TGFβ-induced epithelial-mesenchymal transition: implications for fibrosis and cancer. Clin Transl Med.

[65] Bandzerewicz A, Gadomska-Gajadhur A (2022). Into the tissues: extracellular matrix and its artificial substitutes: cell signalling mechanisms. Cells.

[66] Byron A, Humphries JD, Humphries MJ (2013). Defining the extracellular matrix using proteomics. Int J Exp Pathol.

[67] Macri L, Silverstein D, Clark RAF (2007). Growth factor binding to the pericellular matrix and its importance in tissue engineering. Adv Drug Deliv Rev.

[68] Yurchenco PD (2011). Basement membranes: cell scaffoldings and signaling platforms. Cold Spring Harb Perspect Biol.

[69] Ritty TM, Roth R, Heuser JE (2003). tendon cell array isolation reveals a previously unknown fibrillin-2-containing macromolecular assembly. Structure.

[70] Cui N, Hu M, Khalil RA. Biochemical and biological attributes of matrix metalloproteinases. Prog Mol Biol Transl Sci. 2017;147:1–73. 10.1016/bs.pmbts.2017.02.005 PMC5430303 28413025

[71] Vadasz Z, Kessler O, Akiri G, Gengrinovitch S, Kagan HM, Baruch Y (2005). Abnormal deposition of collagen around hepatocytes in Wilson’s disease is associated with hepatocyte specific expression of lysyl oxidase and lysyl oxidase like protein-2. J Hepatol.

[72] Kagan HM, Li W (2003). Lysyl oxidase: Properties, specificity, and biological roles inside and outside of the cell. J Cell Biochem.

[73] Kim YM, Kim EC, Kim Y (2011). The human lysyl oxidase-like 2 protein functions as an amine oxidase toward collagen and elastin. Mol Biol Rep.

[74] Gill SE, Parks WC (2008). Metalloproteinases and Their Inhibitors: Regulators of Wound Healing. Int J Biochem Cell Biol.

[75] Dunsmore SE, Saarialho-Kere UK, Roby JD, Wilson CL, Matrisian LM, Welgus HG (1998). Matrilysin expression and function in airway epithelium. J Clin Invest.

[76] Atkinson JJ, Toennies HM, Holmbeck K, Senior RM (2007). Membrane type 1 matrix metalloproteinase is necessary for distal airway epithelial repair and keratinocyte growth factor receptor expression after acute injury. Am J Physiol Lung Cell Mol Physiol.

[77] Kim KH, Burkhart K, Chen P, Frevert CW, Randolph-Habecker J, Hackman RC (2005). Tissue Inhibitor of Metalloproteinase-1 Deficiency Amplifies Acute Lung Injury in Bleomycin-Exposed Mice. Am J Respir Cell Mol Biol.

[78] Corry DB, Rishi K, Kanellis J, Kiss A, Song L zhen, Xu J (2002). Decreased allergic lung inflammatory cell egression and increased susceptibility to asphyxiation in MMP2-deficiency. Nat Immunol.

[79] Corry DB, Kiss A, Song L-Z, Song L, Xu J, Lee S-H (2004). Overlapping and independent contributions of MMP2 and MMP9 to lung allergic inflammatory cell egression through decreased CC chemokines. FASEB J.

[80] McGowan SE (1992). Extracellular matrix and the regulation of lung development and repair. FASEB J.

[81] Zhou Y, Horowitz JC, Naba A, Ambalavanan N, Atabai K, Balestrini J (2018). Extracellular matrix in lung development, homeostasis and disease. Matrix Biol.

[82] Bateman ED, Turner-Warwick M, Adelmann-Grill BC (1981). Immunohistochemical study of collagen types in human foetal lung and fibrotic lung disease. Thorax.

[83] Ryu J, Vicencio AG, Yeager ME, Kashgarian M, Haddad GG, Eickelberg O (2005). Differential expression of matrix metalloproteinases and their inhibitors in human and mouse lung development. Thromb Haemost.

[84] Mariani TJ, Sandefur S, Pierce RA (1997). Elastin in Lung Development. Exp Lung Res.

[85] Mouw JK, Ou G, Weaver VM (2014). Extracellular matrix assembly: a multiscale deconstruction. Nat Rev Mol Cell Biol.

[86] Wagenseil JE, Mecham RP (2007). New insights into elastic fiber assembly. Birth Defects Res C Embryo Today.

[87] Burgstaller G, Oehrle B, Gerckens M, White ES, Schiller HB, Eickelberg O (2017). The instructive extracellular matrix of the lung: basic composition and alterations in chronic lung disease. Eur Respir J.

[88] Gaggar A, Weathington N (2016). Bioactive extracellular matrix fragments in lung health and disease. J Clin Investig.

[89] Saikia P, Medeiros CS, Thangavadivel S, Wilson SE (2018). Basement membranes in the cornea and other organs that commonly develop fibrosis. Cell Tissue Res.

[90] Varghese B, Ling Z, Ren X (2022). Reconstructing the pulmonary niche with stem cells: a lung story. Stem Cell Res Ther.

[91] Izvolsky KI, Shoykhet D, Yang Y, Yu Q, Nugent MA, Cardoso WV (2003). Heparan sulfate–FGF10 interactions during lung morphogenesis. Dev Biol.

[92] Patel VN, Pineda DL, Hoffman MP (2017). The function of heparan sulfate during branching morphogenesis. Matrix Biol.

[93] Luo Y, Li N, Chen H, Fernandez GE, Warburton D, Moats R (2018). Spatial and temporal changes in extracellular elastin and laminin distribution during lung alveolar development. Sci Rep.

[94] Rippa AL, Alpeeva EV, Vasiliev AV, Vorotelyak EA (2021). Alveologenesis: What governs secondary septa formation. Int J Mol Sci.

[95] Mohan V, Das A, Sagi I (2020). Emerging roles of ECM remodeling processes in cancer. Semin Cancer Biol.

[96] Wight TN, Potter-Perigo S (2011). The extracellular matrix: an active or passive player in fibrosis?. Am J Physiol Gastrointest Liver Physiol.

[97] Herrera J, Henke CA, Bitterman PB (2018). Extracellular matrix as a driver of progressive fibrosis. J Clin Investig.

[98] Kaplan RN, Riba RD, Zacharoulis S, Bramley AH, Vincent L, Costa C (2005). VEGFR1-positive haematopoietic bone marrow progenitors initiate the pre-metastatic niche. Nature.

[99] Hiratsuka S, Watanabe A, Aburatani H, Maru Y (2006). Tumour-mediated upregulation of chemoattractants and recruitment of myeloid cells predetermines lung metastasis. Nat Cell Biol.

[100] Psaila B, Lyden D (2009). The metastatic niche: adapting the foreign soil. Nat Rev Cancer.

[101] Han X, Aslanian A, Yates JR (2008). Mass spectrometry for proteomics. Curr Opin Chem Biol.

[102] Simunovic F, Winninger O, Strassburg S, Koch HG, Finkenzeller G, Stark GB (2019). Increased differentiation and production of extracellular matrix components of primary human osteoblasts after cocultivation with endothelial cells: a quantitative proteomics approach. J Cell Biochem.

[103] Gocheva V, Naba A, Bhutkar A, Guardia T, Miller KM, Li CMC (2017). Quantitative proteomics identify Tenascin-C as a promoter of lung cancer progression and contributor to a signature prognostic of patient survival. Proc Natl Acad Sci U S A.

[104] Decaris ML, Gatmaitan M, FlorCruz S, Luo F, Li K, Holmes WE (2014). Proteomic analysis of altered extracellular matrix turnover in bleomycin-induced pulmonary fibrosis. Mol Cell Proteomics.

[105] Mann M (2006). Functional and quantitative proteomics using SILAC. Nat Rev Mol Cell Biol.

[106] Dermit M, Dodel M, Mardakheh FK (2017). Methods for monitoring and measurement of protein translation in time and space. Mol BioSyst.

[107] Ong SE, Blagoev B, Kratchmarova I, Kristensen DB, Steen H, Pandey A (2002). Stable isotope labeling by amino acids in cell culture, silac, as a simple and accurate approach to expression proteomics *. Mol Cell Proteomics.

[108] Calve S, Witten AJ, Ocken AR, Kinzer-Ursem TL (2016). Incorporation of non-canonical amino acids into the developing murine proteome. Sci Rep.

[109] Bagert JD, Xie YJ, Sweredoski MJ, Qi Y, Hess S, Schuman EM (2014). Quantitative, time-resolved proteomic analysis by combining bioorthogonal noncanonical amino acid tagging and pulsed stable isotope labeling by amino acids in cell culture. Mol Cell Proteomics.

[110] Fornasiero EF, Mandad S, Wildhagen H, Alevra M, Rammner B, Keihani S (2018). Precisely measured protein lifetimes in the mouse brain reveal differences across tissues and subcellular fractions. Nat Commun.

[111] Rosmark O, Åhrman E, Müller C, Elowsson Rendin L, Eriksson L, Malmström A (2018). Quantifying extracellular matrix turnover in human lung scaffold cultures. Sci Rep.

[112] Rendin LE, Löfdahl A, Åhrman E, Müller C, Notermans T, Michaliková B (2019). Matrisome properties of scaffolds direct fibroblasts in idiopathic pulmonary fibrosis. Int J Mol Sci.

[113] Steward KF, Eilers B, Tripet B, Fuchs A, Dorle M, Rawle R (2020). Metabolic implications of using BioOrthogonal Non-Canonical Amino Acid Tagging (BONCAT) for tracking protein synthesis. Front Microbiol.

[114] Saleh AM, Wilding KM, Calve S, Bundy BC, Kinzer-Ursem TL (2019). Non-canonical amino acid labeling in proteomics and biotechnology. J Biol Eng.

[115] Lee KJ, Kang D, Park HS (2019). Site-specific labeling of proteins using unnatural amino acids. Mol Cells.

[116] van Bergen W, Heck AJR, Baggelaar MP (2022). Recent advancements in mass spectrometry–based tools to investigate newly synthesized proteins. Curr Opin Chem Biol.

[117] Kolb HC, Finn MG, Sharpless KB (2001). Click chemistry: diverse chemical function from a few good reactions. Angew Chem Int Ed.

[118] Landgraf P, Antileo ER, Schuman EM, Dieterich DC (2015). BONCAT: metabolic labeling, click chemistry, and affinity purification of newly synthesized proteomes.

[119] Dieterich DC, Link AJ, Graumann J, Tirrell DA, Schuman EM (2006). Selective identification of newly synthesized proteins in mammalian cells using bioorthogonal noncanonical amino acid tagging (BONCAT). Proc Natl Acad Sci U S A.

[120] Koren SA, Gillett DA, D’alton SV, Hamm MJ, Abisambra JF (2019). Proteomic techniques to examine neuronal translational dynamics. Int J Mol Sci.

[121] Howden AJM, Geoghegan V, Katsch K, Efstathiou G, Bhushan B, Boutureira O (2013). QuaNCAT: quantitating proteome dynamics in primary cells. Nature Methods.

[122] Ma Y, Mcclatchy DB, Barkallah S, Wood WW, Yates JR (2017). HILAQ: A novel strategy for newly synthesized protein quantification. J Proteome Res.

[123] McLeod CM, Mauck RL (2016). High fidelity visualization of cell-to-cell variation and temporal dynamics in nascent extracellular matrix formation. Sci Rep.

[124] Loebel C, Mauck RL, Burdick JA (2019). Local nascent protein deposition and remodelling guide mesenchymal stromal cell mechanosensing and fate in three-dimensional hydrogels. Nat Mater.

[125] Jacobson KR, Saleh AM, Lipp SN, Ocken AR, Kinzer-Ursem TL, Calve S. Extracellular matrix protein composition dynamically changes during murine forelimb development. bioRxiv. 2020. 10.1101/2020.06.17.158204. 10.1016/j.isci.2024.108838 PMC10831947 38303699

[126] Saleh AM, Jacobson KR, Kinzer-Ursem TL, Calve S (2019). Dynamics of non-canonical amino acid-labeled intra- and extracellular proteins in the developing mouse. Cell Mol Bioeng.

[127] Schiapparelli LM, McClatchy DB, Liu HH, Sharma P, Yates JR, Cline HT (2014). Direct detection of biotinylated proteins by mass spectrometry. J Proteome Res.

[128] Ullrich M, Liang V, Chew YL, Banister S, Song X, Zaw T (2014). Bio-orthogonal labeling as a tool to visualize and identify newly synthesized proteins in Caenorhabditis elegans. Nat Protoc.

[129] Hinz FI, Dieterich DC, Tirrell DA, Schuman EM (2012). Noncanonical amino acid labeling in vivo to visualize and affinity purify newly synthesized proteins in larval zebrafish. ACS Chem Neurosci.

[130] Changa PV, Preschera JA, Sletten EM, Baskin JM, Miller IA, Agard NJ (2010). Copper-free click chemistry in living animals. Proc Natl Acad Sci U S A.

[131] Chang PV, Chen X, Smyrniotis C, Xenakis A, Hu T, Bertozzi CR (2009). Metabolie labeling of sialic acids in living animals with alkynyl sugars. Angew Chem Int Ed Engl.

[132] Lynch M, Barallobre-Barreiro J, Jahangiri M, Mayr M (2016). Vascular proteomics in metabolic and cardiovascular diseases. J Intern Med.

[133] Zaro BW, Hang HC, Pratt MR (2013). Incorporation of unnatural sugars for the identification of glycoproteins. Methods Mol Biol.

[134] Spiciarich DR, Nolley R, Maund SL, Purcell SC, Herschel J, Iavarone AT (2017). Bioorthogonal labeling of human prostate cancer tissue slice cultures for glycoproteomics. Angew Chem Int Ed.

[135] Colley KJ, Varki A, Haltiwanger RS, et al. Cellular organization of glycosylation. In: Varki A, Cummings RD, Esko JD, et al., editors. Essentials of Glycobiology (4th edition). New York: Cold Spring Harbor; 2022. p. 41–50.

[136] Ren X, Evangelista-Leite D, Wu T, Rajab KT, Moser PT, Kitano K (2018). Metabolic glycan labeling and chemoselective functionalization of native biomaterials. Biomaterials.

[137] Saad OM, Leary JA (2003). Compositional analysis and quantification of heparin and heparan sulfate by electrospray ionization ion trap mass spectrometry. Anal Chem.

[138] Zaia J, Costello CE (2000). Compositional analysis of glycosaminoglycans by electrospray mass spectrometry. Anal Chem.

[139] Desaire H, Leary JA (2000). Detection and quantification of the sulfated disaccharides in chondroitin sulfate by electrospray tandem mass spectrometry. J Am Soc Mass Spectrom.

[140] Juhasz P, Biemann K (1995). Utility of non-covalent complexes in the matrix-assisted laser desorption ionization mass spectrometry of heparin-derived oligosaccharides. Carbohyd Res.

[141] Kuberan B, Lech M, Zhang L, Wu ZL, Beeler DL, Rosenberg RD (2002). Analysis of heparan sulfate oligosaccharides with ion pair-reverse phase capillary high performance liquid chromatography-microelectrospray ionization time-of-flight mass spectrometry. J Am Chem Soc.

[142] Zhang X, Zhang Y (2013). Applications of azide-based bioorthogonal click chemistry in glycobiology. Molecules.

[143] Chung CZ, Amikura K, Söll D (2020). Using genetic code expansion for protein biochemical studies. Front Bioeng Biotechnol.

[144] Wang L, Xie J, Schultz PG (2006). Expanding the genetic code. Annu Rev Biophys Biomol Struct.

[145] Do AV, Khorsand B, Geary SM, Salem AK (2015). 3D printing of scaffolds for tissue regeneration applications. Adv Healthcare Mater.

[146] Sears NA, Seshadri DR, Dhavalikar PS, Cosgriff-Hernandez E (2016). A review of three-dimensional printing in tissue engineering. Tissue Engineering - Part B: Reviews.

[147] Bittner SM, Guo JL, Melchiorri A, Mikos AG (2018). Three-dimensional printing of multilayered tissue engineering scaffolds. Mater Today.

[148] Patra S, Young V (2016). A review of 3D printing techniques and the future in biofabrication of bioprinted tissue. Cell Biochem Biophys.

[149] An J, Teoh JEM, Suntornnond R, Chua CK (2015). Design and 3D printing of scaffolds and tissues. Engineering.

[150] Murphy SV, Atala A (2014). 3D bioprinting of tissues and organs. Nat Biotechnol.

[151] Temple JP, Hutton DL, Hung BP, Huri PY, Cook CA, Kondragunta R (2014). Engineering anatomically shaped vascularized bone grafts with hASCs and 3D-printed PCL scaffolds. J Biomed Mater Res A.

[152] Kim WJ, Kim M, Kim GH (2018). 3D-printed biomimetic scaffold simulating microfibril muscle structure. Adv Func Mater.

[153] Grigoryan B, Paulsen SJ, Corbett DC, Sazer DW, Fortin CL, Zaita AJ (1979). Multivascular networks and functional intravascular topologies within biocompatible hydrogels. Science.

[154] Lee A, Hudson AR, Shiwarski DJ, Tashman JW, Hinton TJ, Yerneni S (1979). 3D bioprinting of collagen to rebuild components of the human heart. Science.

[155] Khani N, Nadernezhad A, Bartolo P, Koc B (2017). Hierarchical and spatial modeling and bio-additive manufacturing of multi-material constructs. CIRP Ann.

[156] Liu W, Zhang YS, Heinrich MA, de Ferrari F, Jang HL, Bakht SM (2017). Rapid continuous multimaterial extrusion bioprinting. Adv Mater.

[157] Rocca M, Fragasso A, Liu W, Heinrich MA, Zhang YS (2018). Embedded multimaterial extrusion bioprinting. SLAS Technol.

[158] Grigoryan B, Sazer DW, Avila A, Albritton JL, Padhye A, Ta AH (2021). Development, characterization, and applications of multi-material stereolithography bioprinting. Sci Rep.

[159] Choudhury D, Tun HW, Wang T, Naing MW (2018). Organ-derived decellularized extracellular matrix: a game changer for bioink manufacturing?. Trends Biotechnol.

[160] Kim BS, Das S, Jang J, Cho D-W (2020). decellularized extracellular matrix-based bioinks for engineering tissue- and organ-specific microenvironments. Chem Rev.

[161] Mostaco-Guidolin LB, Loube J, Barlow A, Osei ET, Vasilescu DM, Hsieh A (2021). Second harmonic generation imaging of collagen scaffolds within the alveolar ducts of healthy and emphysematous mouse lungs. Histochem Cell Biol.

[162] Lipp SN, Jacobson KR, Hains DS, Schwarderer AL, Calve S (2021). 3D mapping reveals a complex and transient interstitial matrix during murine kidney development. J Am Soc Nephrol.

[163] Mayorca-Guiliani AE, Willacy O, Madsen CD, Rafaeva M, Elisabeth Heumüller S, Bock F (2019). Decellularization and antibody staining of mouse tissues to map native extracellular matrix structures in 3D. Nat Protoc.

[164] Mayorca-Guiliani AE, Madsen CD, Cox TR, Horton ER, Venning FA, Erler JT (2017). ISDoT: in situ decellularization of tissues for high-resolution imaging and proteomic analysis of native extracellular matrix. Nat Med.

[165] Angel PM, Schwamborn K, Comte‐Walters S, Clift CL, Ball LE, Mehta AS (2019). Extracellular matrix imaging of breast tissue pathologies by MALDI–Imaging Mass Spectrometry. Proteomics Clin Appl.

[166] Piehowski PD, Zhu Y, Bramer LM, Stratton KG, Zhao R, Orton DJ (2020). Automated mass spectrometry imaging of over 2000 proteins from tissue sections at 100-μm spatial resolution. Nat Commun.

[167] Stickels RR, Murray E, Kumar P, Li J, Marshall JL, di Bella DJ (2021). Highly sensitive spatial transcriptomics at near-cellular resolution with Slide-seqV2. Nat Biotechnol.

[168] Loebel C, Kwon MY, Wang C, Han L, Mauck RL, Burdick JA (2020). Metabolic labeling to probe the spatiotemporal accumulation of matrix at the chondrocyte-hydrogel interface. Adv Func Mater.

[169] Miri AK, Mirzaee I, Hassan S, Mesbah Oskui S, Nieto D, Khademhosseini A (2019). Effective bioprinting resolution in tissue model fabrication. Lab Chip.

[170] LeBleu VS, MacDonald B, Kalluri R (2007). Structure and function of basement membranes. Exp Biol Med.

[171] Doerschuk CM, Beyers N, Coxson HO, Wiggs B, Hogg JC (1993). Comparison of neutrophil and capillary diameters and their relation to neutrophil sequestration in the lung. J Appl Physiol.

[172] Ren B, Chen X, Ma Y, Du S, Qian S, Xu Y (2018). Dynamical release nanospheres containing cell growth factor from biopolymer hydrogel via reversible covalent conjugation. J Biomater Sci Polym Ed.

[173] Ye L, Wu X, Mu Q, Chen B, Duan Y, Geng X (2011). Heparin-conjugated PCL scaffolds fabricated by electrospinning and loaded with fibroblast growth factor 2. J Biomater Sci Polym Ed.

[174] Gelmi A, Schutt CE (2021). Stimuli-responsive biomaterials: scaffolds for stem cell control. Adv Healthcare Mater.

[175] So WH, Wong CTT, Xia J (2018). Peptide photocaging: a brief account of the chemistry and biological applications. Chin Chem Lett.

[176] Wylie RG, Ahsan S, Aizawa Y, Maxwell KL, Morshead CM, Shoichet MS (2011). Spatially controlled simultaneous patterning of multiple growth factors in three-dimensional hydrogels. Nat Mater.

[177] Guvendiren M, Burdick JA (2012). Stiffening hydrogels to probe short- and long-term cellular responses to dynamic mechanics. Nat Commun.

[178] McKinnon DD, Brown TE, Kyburz KA, Kiyotake E, Anseth KS (2014). Design and characterization of a synthetically accessible, photodegradable hydrogel for user-directed formation of neural networks. Biomacromol.

[179] Gjorevski N, Nikolaev M, Brown TE, Mitrofanova O, Brandenberg N, DelRio FW (1979). Tissue geometry drives deterministic organoid patterning. Science.

[180] Hussey GS, Dziki JL, Lee YC, Bartolacci JG, Behun M, Turnquist HR (2019). Matrix bound nanovesicle-associated IL-33 activates a pro-remodeling macrophage phenotype via a non-canonical, ST2-independent pathway. J Immunol Regen Med.

[181] Huleihel L, Bartolacci JG, Dziki JL, Vorobyov T, Arnold B, Scarritt ME (2017). Matrix-bound nanovesicles recapitulate extracellular matrix effects on macrophage phenotype. Tissue Engineering - Part A.

[182] van der Merwe Y, Faust AE, Steketee MB (2017). Matrix bound vesicles and miRNA cargoes are bioactive factors within extracellular matrix bioscaffolds. Neural Regen Res.

[183] Simeone P, Bologna G, Lanuti P, Pierdomenico L, Guagnano MT, Pieragostino D (2020). Extracellular vesicles as signaling mediators and disease biomarkers across biological barriers. Int J Mol Sci.

[184] Hussey GS, Molina CP, Cramer MC, Tyurina YY, Tyurin VA, Lee YC (2020). Lipidomics and RNA sequencing reveal a novel subpopulation of nanovesicle within extracellular matrix biomaterials. Sci Adv.

[185] Dankovich TM, Kaushik R, Olsthoorn LHM, Petersen GC, Giro PE, Kluever V (2021). Extracellular matrix remodeling through endocytosis and resurfacing of Tenascin-R. Nat Commun.

